# Strategies for modelling the intestinal mucus interface: Focus on drug transport and host-microbiota interactions

**DOI:** 10.1016/j.ijpx.2026.100672

**Published:** 2026-09-26

**Authors:** Aixa Aguilera-Garrido, Agathe Lemée, Julien Claudel, Halima Alem

**Affiliations:** a Université de Lorraine-CNRS, IJL, 54000 Nancy, France; b Institut Universitaire de France, 75000 Paris, France

**Keywords:** Mucus, Intestinal barrier, Mucus model

## Abstract

The intestinal mucus barrier is a complex, dynamic hydrogel that plays a central role in host–microbiota interactions, nutrient transport, and protection against pathogens. Understanding its physicochemical properties has become essential for developing predictive in vitro and in silico models for drug delivery, microbiome research, and disease modelling. Over the past decade, advances in polymer chemistry, glycoscience, and microfluidics have enabled the development of increasingly sophisticated mucus models that mimic structural, rheological, and biochemical features of native intestinal mucus. This review provides a comprehensive overview of recent progress in gut-mucus chemical model development, focusing on mucin biochemistry, hydrogel-based synthetic systems, microphysiological platforms, and an introduction on computational modelling approaches. Special emphasis is placed on emerging strategies to reproduce mucus heterogeneity, viscoelasticity, and microbial interactions. We critically assess the strengths and limitations of current models and highlight future directions for improving physiological relevance, including multi-scale integration and personalized mucus modelling. This review aims to provide a framework for researchers developing next-generation mucus models for biomedical and pharmaceutical applications.

## Highlights

• Intestinal mucus models differ markedly in origin and physiological relevance.
• Mucus properties strongly influence drug transport and formulation performance.
• Cell-derived and microphysiological models improve biological representation.
• Mucus connects dietary glycans, microbiota metabolism and immune signalling.
• A fit-for-purpose framework guides the selection of experimental mucus models.

## Graphical abstract

**Figure:**
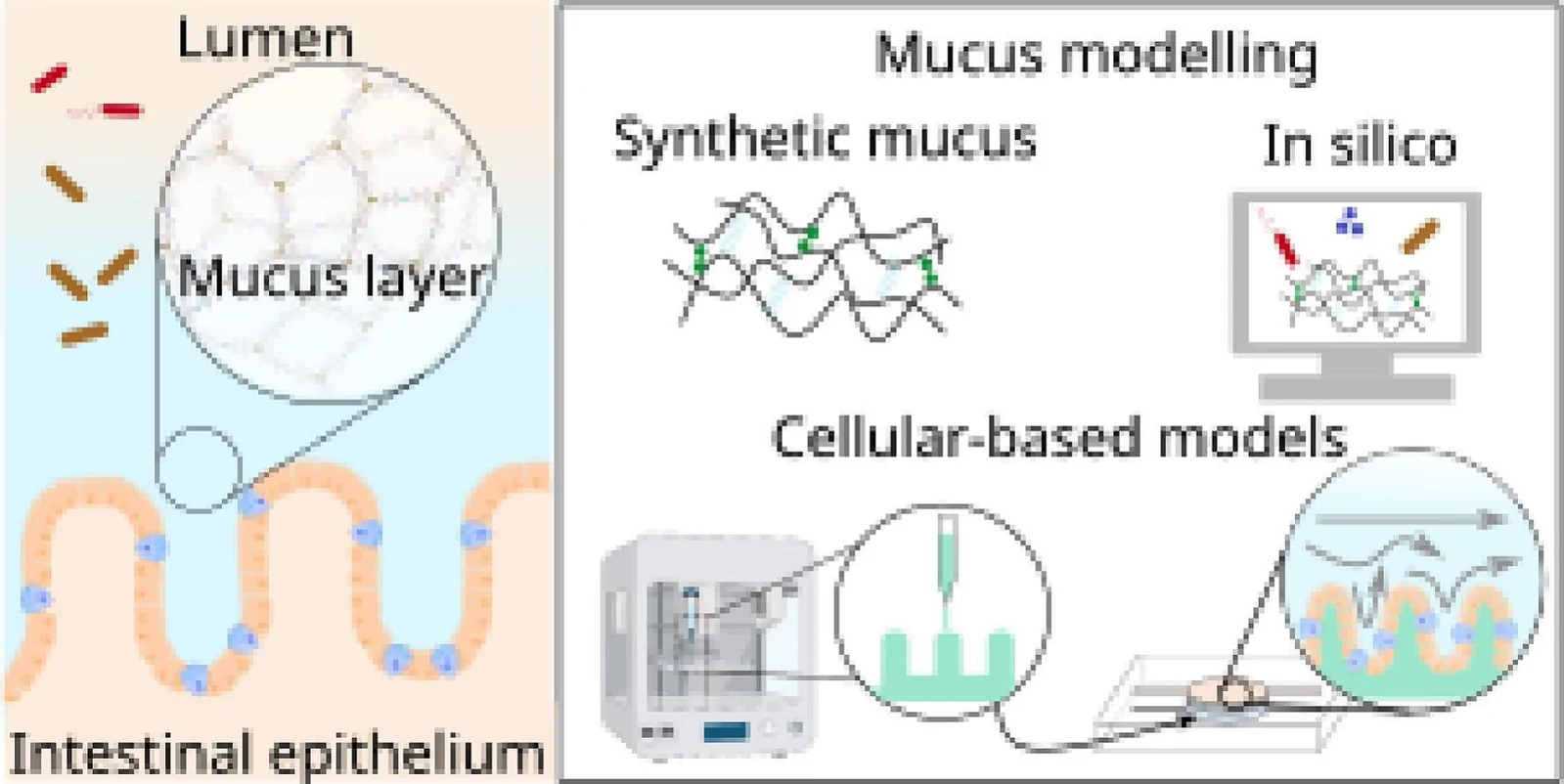

## 1 Introduction

The gastrointestinal tract is lined by a highly specialized mucus layer that plays a central role in maintaining intestinal homeostasis (Paone and Cani, 2020). Acting as both a physical and biochemical barrier, intestinal mucus protects the underlying epithelium from mechanical stress, pathogens, and toxic compounds, while simultaneously allowing the selective passage of nutrients, metabolites, and signalling molecules (Paone and Cani, 2020). Over the past decade, this hydrated gel has emerged as a key regulator of host–microbiota interactions and a critical determinant of drug transport and bioavailability. As a result, there has been growing interest in understanding its structure, composition, and function, as well as in developing reliable models that can reproduce its complex behaviour in vitro (Luis and Hansson, 2023a).

Intestinal mucus is primarily composed of mucins, a family of high-molecular-weight glycoproteins produced by goblet cells and characterized by extensive O-linked glycosylation. Among these, MUC2 is the predominant gel-forming mucin in the intestine and is responsible for the formation of a three-dimensional polymeric network that gives mucus its viscoelastic properties (Luis and Hansson, 2023).

The dense glycosylation of mucins, which provides then with their characteristic ‘bottlebrush’ structure, not only contributes to hydration and steric stabilization but also generates a chemically diverse and highly dynamic interface. The glycan moieties attached to mucins vary in composition, charge, and branching, leading to significant heterogeneity across different regions of the gastrointestinal tract and in response to physiological or pathological conditions. This complexity is further amplified by the presence of lipids, proteins, ions, and microbial components, all of which contribute to the emergent properties of mucus as a functional biomaterial (Round et al., 2012).

On one hand from a physicochemical perspective, intestinal mucus behaves as a non-Newtonian viscoelastic hydrogel (Fig. 2D). Its ability to undergo reversible deformation under shear stress is essential for accommodating peristaltic motion and the continuous flow of luminal contents (Klitgaard et al., 2024). At the same time, the structural integrity of the mucin network ensures the maintenance of a protective barrier. The rheological properties of mucus are highly sensitive to environmental factors such as pH, ionic strength, and the presence of multivalent ions, which can modulate intermolecular interactions and network organization.

On the other hand, the mucus layer does not have the same organization along the whole digestive system. The mucus layer on the small intestine consists of a single, easily removable and penetrable layer. Here, the bacteria are kept away from the epithelium thank to the release of antibacterial mediators. Differently, mucus exhibits a distinct bilayered architecture in the colon, consisting of a dense inner layer that is largely impermeable to bacteria and a looser outer layer that serves as a habitat for commensal microbiota (Fig. 1). This spatial organization is crucial for balancing microbial colonization with host protection. The growing recognition of mucus as an active biological interface has important implications for both fundamental research and applied sciences. In the context of microbiology, mucus serves as a key mediator of host–microbe interactions, influencing microbial adhesion, nutrient availability, and immune signalling. Certain commensal bacteria can degrade mucin glycans, thereby glycans composition shapes the composition and function of the microbial community (Schroeder, 2019). Conversely, alterations in mucus structure or composition have been associated with various diseases, including inflammatory bowel disease, colorectal cancer, and metabolic disorders (Fig. 1). Understanding these interactions requires experimental systems that can capture the dynamic and heterogeneous nature of mucus (Schroeder, 2019).

**Fig. 1:**
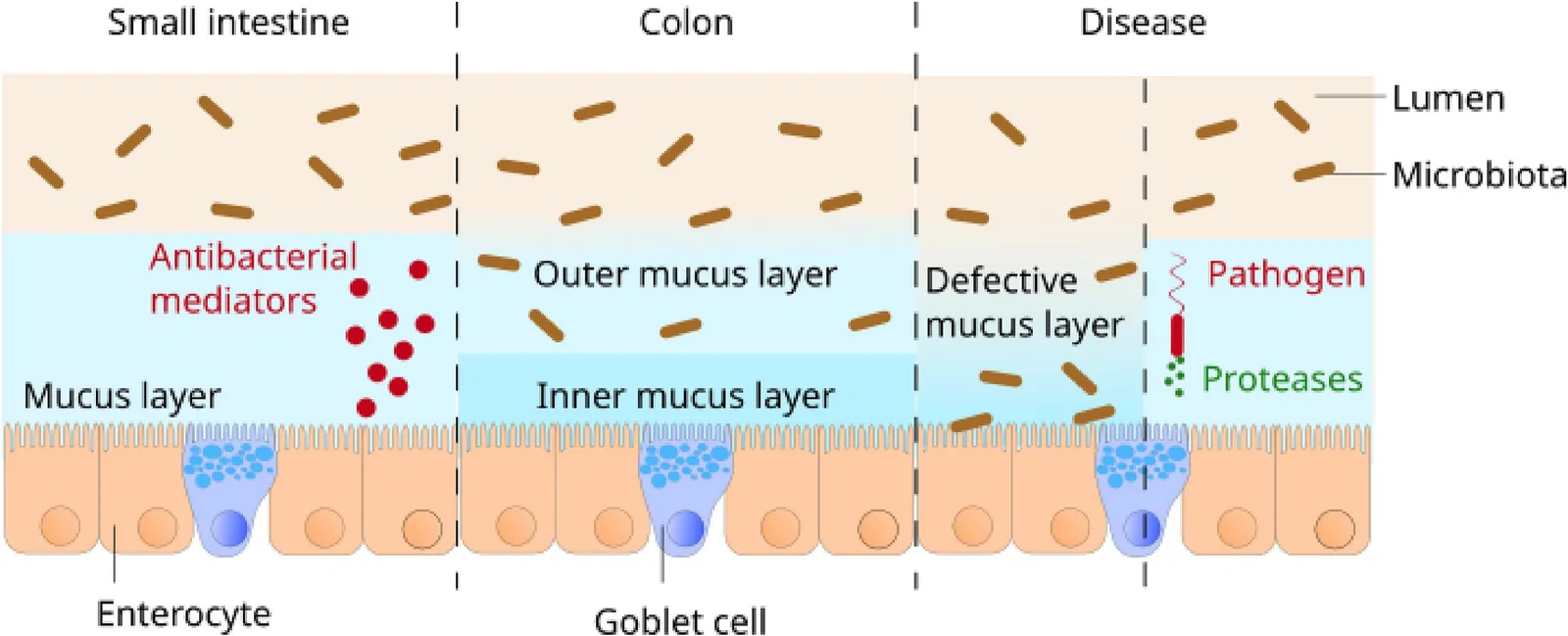
Mucus layer structure in the small intestine, in the colon and the role of mucus as defensive barrier against pathogens. In the small intestine mucus is easy penetrable to ease nutrient absorption. Here, bacteria colonization of epithelium is prevented by releasing immune molecules. In the colon, the inner compacted mucus layer avoids microorganisms from reaching the epithelium. However, diseased states can be produced as a consequence of a defective mucus layer that allow microorganisms penetration, or by microorganisms-driven mucus disruption and colonization.

In pharmaceutical science, mucus is a significant barrier to the delivery of therapeutic agents, particularly for orally administered drugs and nanoparticle-based systems. The diffusion of molecules through mucus depends on a combination of size, charge, hydrophobicity, and surface chemistry, as well as on the structural properties of the mucus network itself (Lock et al., 2018). Traditional in vitro models, often based on simple mucin solutions, fail to accurately reproduce these parameters, leading to discrepancies between experimental results and in vivo outcomes. Consequently, there is a pressing need for advanced mucus models that can provide more predictive insights into drug transport and bioavailability.

Recent years have seen the emergence of a wide range of experimental and theoretical approaches aimed at addressing these challenges (Macedo et al., 2023; Macierzanka et al., 2019; Pinholt et al., 2021; Witten et al., 2018; Xu et al., 2021). In the field of materials science, synthetic and semi-synthetic hydrogels have been developed to mimic the viscoelastic and transport properties of mucus. These systems offer tunable mechanical and chemical characteristics, enabling systematic investigation of structure/function relationships. Hybrid models that combine natural mucins with synthetic polymers have further improved the balance between biological relevance and reproducibility. At the same time, advances in glycoengineering have enabled the design of mucin-like glycoproteins with controlled glycan structures, providing new opportunities to study the role of glycosylation in mucus function (Bergstrom and Xia, 2022; Butenhof and Gerken, 1993; Wu et al., 2023).

Parallel to these developments, microphysiological systems such as gut-on-chip platforms have introduced a new level of complexity by incorporating living cells, fluid flow, and mechanical forces (De Rudder et al., 2026; Movcana et al., 2026; Özkan et al., 2024; Wang et al., 2025). These devices allow the formation of endogenous mucus layers under dynamic conditions and enable the study of host–microbe interactions in a controlled environment. In addition, computational modelling has become an increasingly important tool for understanding mucus behaviour across multiple scales. Molecular simulations provide insights into mucin conformation and interactions, while continuum models describe macroscopic transport phenomena such as diffusion and flow (Valibeknejad et al., 2023; Zhao et al., 2024). The integration of these approaches is beginning to yield predictive frameworks that can guide the design of more realistic mucus models.

Despite these advances, significant challenges remain. Replicating the full compositional and structural heterogeneity of native mucus, particularly in terms of glycan diversity and spatial organization, is still beyond the reach of most current models. Moreover, capturing the dynamic turnover of mucus and its interactions with the microbiota requires the integration of chemical, biological, and mechanical components in a single system. Addressing these challenges will require continued interdisciplinary collaboration and innovation.

In this review, we provide a comprehensive overview of recent progress in the development of chemical models of intestinal mucus. We begin by examining the fundamental structure and physicochemical properties of mucus, with a particular focus on mucin biochemistry and network organization. We then discuss the various experimental approaches used to model mucus, including purified mucin systems, synthetic hydrogels, hybrid materials, and microphysiological platforms. Finally, we explore the interactions between mucus, microbiota, and therapeutic agents, as well as the role of computational modelling in advancing our understanding of this complex system. By highlighting both achievements and remaining challenges, this review aims to provide a framework for the design of next-generation mucus models with improved physiological relevance and predictive power.

## 2 Structure, composition et physico-chemical properties of the intestinal mucus

Mucus complexity arises from a hierarchical organization spanning molecular to macroscopic scales, combining biochemical diversity with unique physicochemical behaviour (Choudhury et al., 2023; Round et al., 2012). At the molecular level, mucins, particularly MUC2 in the intestine, are responsible for the gel-forming properties of mucus. These high-molecular-weight glycoproteins are characterized by a central domain rich in serine, proline and threonine residues that serve as sites for extensive O-linked glycosylation, resulting in densely glycosylated “bottlebrush” architectures (Fig. 2A). Glycans can represent up to 80% of mucin mass, and their structural diversity, comprising neutral, sialylated, and sulfated oligosaccharides, confers critical functional properties such as hydration, electrostatic interactions, and resistance to proteolysis (Wood et al., 2025). Recent advances in glycomics and mass spectrometry have revealed a previously underappreciated heterogeneity in mucin glycosylation patterns, with spatial variation along the gastrointestinal tract and dynamic modulation in response to diet, microbiota, and disease states (Helms et al., 2024). (See Fig. 3.)

**Fig. 2:**
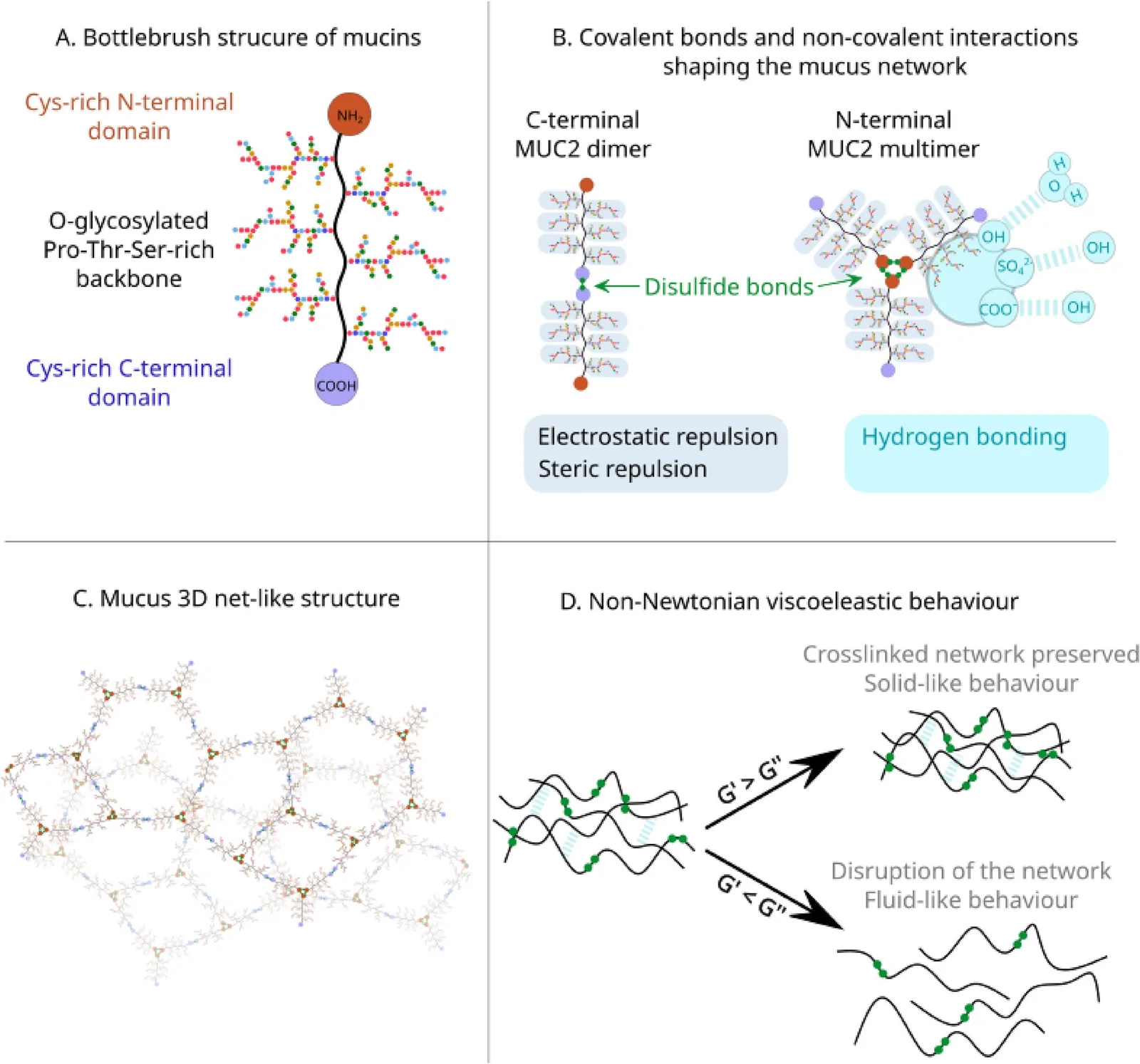
Mucus molecular structure and behaviour. A. Structure of mucins, the main building block of the mucus network. B. Interactions within the mucins network defining the structure of the mucus layer. C. Mucus 3D structure showing mucins interconnexion. D. Strain-Induced transition from intact to disrupted gel network showing the non-Newtonian behaviour of the mucus network. Considering G' the initial shear modulus and G" the shear modulus after shear stress change, for G′ > G”, *the material exhibits solid-like (elastic-dominated) behaviour, whereas for* G′ < G”, a *transition to fluid-like behaviour is observed.*

**Fig. 3:**
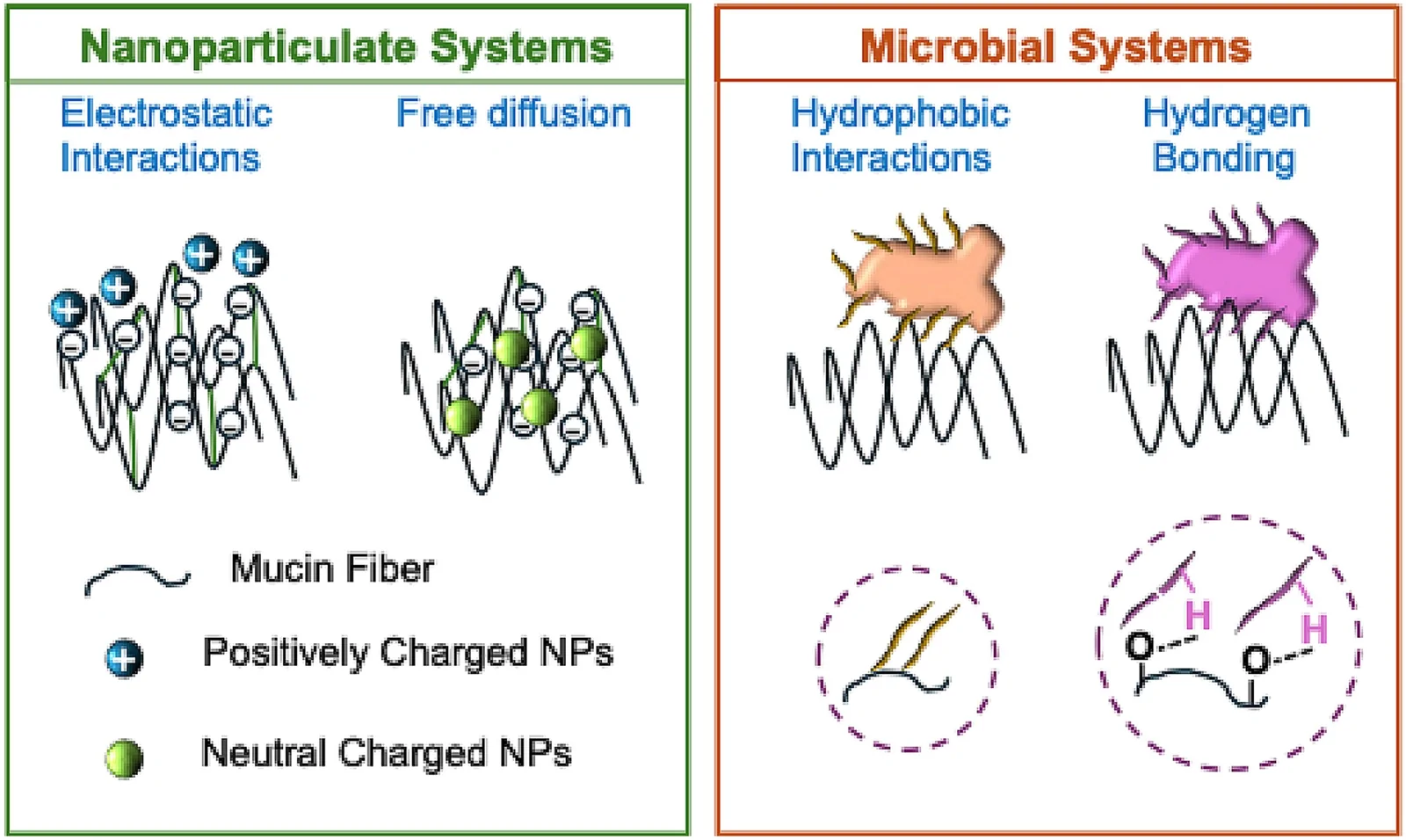
Nanoparticles (NPs) and microbiota interaction with mucus.

Beyond their primary structure, mucins assemble covalently through their N-terminal and C-terminal cysteine-rich domains to form multimers (Fig. 2B). These are stored in a tightly packed way on secretory vesicles in goblet cells. Upon release from goblet cells, the pH increases and the concentration of Ca^2+^ is reduced. Under these conditions, the negative charged glycosylated regions are no longer shielded and the network of mucins is expanded and hydrated (Ambort et al., 2012). Expanded network further stablish non-covalent interactions, forming entangled and crosslinked matrices that define mucus rheology (Fig. 2C). This network is highly hydrated, typically containing more than 95% water, yet exhibits viscoelastic properties that are essential for its biological function. From a rheological perspective, mucus behaves as a non-Newtonian fluid (Fig. 2D), displaying shear-thinning characteristics that facilitate the movement of luminal contents while maintaining barrier integrity under low shear conditions. Advanced rheometry studies have demonstrated that mucus exhibits both elastic (storage modulus) and viscous (loss modulus) components (Bej and Haag, 2022; Klitgaard et al., 2024), with their relative contributions depending on frequency and strain. Importantly, these properties are highly sensitive to environmental factors such as pH, ionic strength, and the presence of divalent cations, which influence mucin conformation and intermolecular interactions.

At the mesoscale, mucus exhibits a heterogeneous porous structure with pore sizes ranging from tens to hundreds of nanometers, depending on location and physiological conditions. This porosity is a key determinant of selective permeability, governing the diffusion of macromolecules, nanoparticles, and microorganisms (Carlson et al., 2018; Witten et al., 2018). Notably, the colon features a stratified mucus organization, with an inner dense layer that is largely impermeable to bacteria and an outer loose layer that serves as a habitat for commensal microbiota. This two-layer architecture has been extensively characterized in recent studies using advanced imaging techniques such as confocal microscopy and cryo-electron tomography (López-Jiménez and Mostowy, 2021; Sun et al., 2020).

Electrostatic interactions play a central role in mucus function, as the negative charge of mucin glycans, arising primarily from sialic acid and sulfate groups, creates a polyanionic environment. This influences the transport of charged species, leading to selective retention or exclusion depending on molecular properties (Witten et al., 2018). For example, positively charged nanoparticles tend to adhere to mucus due to electrostatic attraction, whereas neutral, hydrophilic particles can diffuse easily. Hydrophobic interactions and hydrogen bonding further contribute to the complex interaction landscape within mucus, affecting both molecular transport and microbial adhesion.

Another key aspect of mucus is its dynamic turnover. In vivo, mucus is continuously secreted by goblet cells and degraded by enzymatic activity, resulting in a steady-state balance that maintains barrier function while allowing adaptability. This turnover is particularly important in the context of host–microbe interactions, as certain commensal bacteria possess mucin-degrading enzymes that enable them to utilize mucus glycans as a nutrient source. This metabolic activity can, in turn, influence mucus thickness and composition, creating a feedback loop between the host and microbiota (Schroeder, 2019).

Recent research has also highlighted the role of mucus as an active signalling interface. Mucin glycans can interact with microbial lectins and host receptors, modulating immune responses and microbial colonization patterns. Furthermore, mucus can act as a reservoir for antimicrobial peptides and immunoglobulins, enhancing its protective function. These findings underscore the importance of considering mucus not merely as a passive barrier but as an active participant in gastrointestinal physiology (Bergstrom and Xia, 2022)**.**

Despite significant progress in characterizing mucus, reproducing its complexity in vitro remains challenging. Many traditional models fail to capture key features such as glycan diversity, dynamic turnover, and spatial heterogeneity. As a result, there is a growing need for advanced in vitro models that can accurately mimic these properties. Understanding the fundamental physicochemical principles governing mucus behaviour is therefore essential for guiding the design of such models, as discussed in the following sections.

## 3 Models of the intestinal mucus barrier

The development of physiologically relevant mucus models is complicated by the intrinsic heterogeneity of the gastrointestinal mucus barrier. Its composition, thickness, organization, glycosylation, viscoelasticity, and turnover vary along the gastrointestinal tract and depend on species, age, diet, microbiota composition, and health status (Ermund et al., 2013; Paone and Cani, 2020; Witten et al., 2018). In the small intestine, mucus is generally organized as a single, loose and penetrable layer that facilitates nutrient absorption, while antimicrobial molecules limit bacterial contact with the epithelium. By contrast, colonic mucus comprises a dense inner layer that restricts bacterial penetration and a looser outer layer that provides a habitat and nutrient source for commensal microorganisms (Ermund et al., 2013; Luis and Hansson, 2023c).Inflammation, infection, dysbiosis, medication, and dietary changes can further modify mucus secretion and barrier properties (Luis and Hansson, 2023c; Witten et al., 2018).

No single model can reproduce all these regional, biological, and physicochemical characteristics. Model selection therefore involves a compromise between biological fidelity, experimental control, reproducibility, scalability, and ethical acceptability. (McCoy et al., 2024; Wright et al., 2023) Laboratory models can be classified according to the origin of the mucus into purified animal-derived mucins, native mucus maintained on or removed from intestinal tissue, mucus endogenously secreted by cultured cells, and engineered semi-synthetic or fully synthetic mucus mimics. These materials can subsequently be incorporated into static diffusion assays, epithelial co-cultures, organoids, or dynamic microphysiological systems.

The optimal model should be selected according to the intended application. Studies of defined mucin–formulation interactions do not necessarily require a living epithelial system, whereas investigations of epithelial uptake, microbial colonization, immune signalling, or disease-specific mucus require progressively more biologically complex models. This section describes the principal laboratory-available approaches and their respective domains of application. To facilitate fit-for-purpose model selection, Fig. 4 provides a decision framework linking the intended experimental objective to the most appropriate modelling strategy and indicates whether the mucus component can be artificial, animal-derived, or endogenously secreted by epithelial cells.

**Fig. 4:**
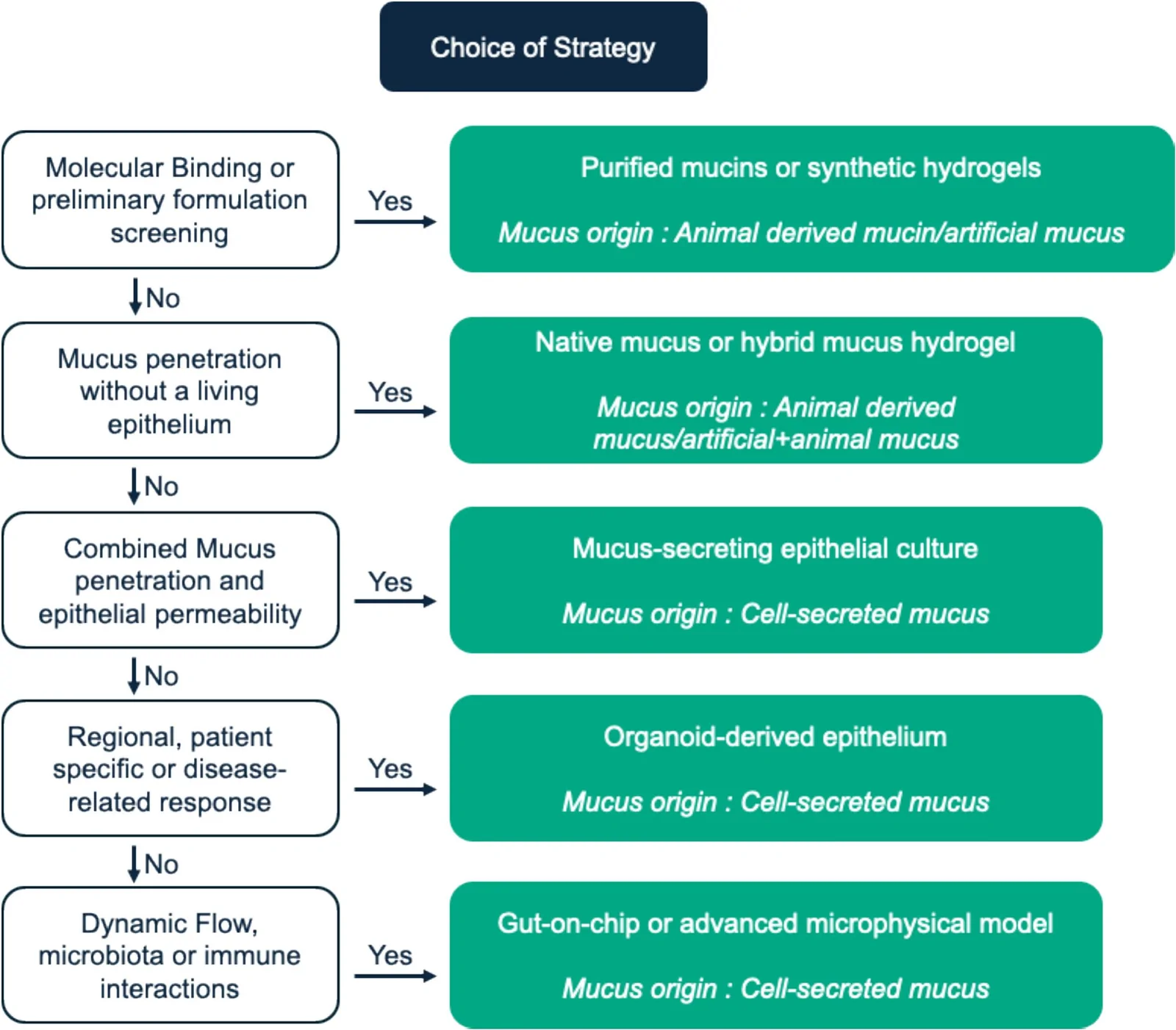
Fit-for-purpose selection of intestinal mucus models according to the research objective and mucus origin.

### 3.1 Purified mucins and reconstituted mucin dispersions

One of the simplest approaches to reproduce the mucus barrier in vitro is the reconstitution of commercially available animal-derived mucins (Lock et al., 2018; Wright et al., 2023). These materials are extracted, purified, lyophilized, and subsequently redispersed in an aqueous buffer at a selected concentration. Porcine gastric mucin and bovine submaxillary mucin are the most frequently used preparations because of their availability, relatively low cost, simple handling, and compatibility with high-throughput assays. (Lock et al., 2018; Wright et al., 2023).

Commercial mucins should not, however, be considered equivalent to native intestinal mucus. Extraction, purification, and lyophilization can fragment high-molecular-weight mucin assemblies and disrupt the covalent and noncovalent interactions responsible for their native supramolecular organization. (Schömig et al., 2016; Wright et al., 2023) Purification also removes lipids, ions, proteins, immunoglobulins, antimicrobial compounds, cellular debris, and other components that contribute to mucus structure and function (Lock et al., 2018). Rehydration cannot fully restore these components or the original organization of the mucin network.

As a result, reconstituted mucin dispersions commonly exhibit rheological properties that differ from those of native mucus (Macierzanka et al., 2019; Schömig et al., 2016; Yildiz et al., 2015). Even at concentrations exceeding physiological mucin levels, commercial preparations may behave predominantly as viscous solutions rather than elastic-dominated hydrogels. Their pore architecture, molecular-weight distribution, glycan presentation, and selective permeability may also differ from those of native intestinal mucus. In addition, porcine gastric and bovine submaxillary mucins differ from intestinal MUC2 in their protein structure, glycosylation, and anatomical function (Schömig et al., 2016).

Despite these limitations, purified mucins remain valuable for controlled and comparative experiments. They are particularly suitable for investigating the adsorption or binding of drugs, polymers, microorganisms, and nanoparticles to mucins. Such interactions can be evaluated through changes in zeta potential, spectroscopy, calorimetry, quartz crystal microbalance measurements, or rheological properties (Mansfield et al., 2015). These assays provide useful initial information on the relative mucoadhesive or mucus-inert behaviour of different formulations.

Reconstituted mucins can also be incorporated into Transwell inserts, diffusion chambers, microfluidic devices, and microscopic tracking assays to compare molecular or particulate permeation (Aguilera-Garrido et al., 2023; Wong et al., 2020). They may additionally be deposited on the apical surface of cells monolayers to introduce a mucus-like barrier into epithelial permeability assays (AlShmmari et al., 2025). However, transport results depend strongly on mucin source, concentration, buffer composition, pH, ionic strength, layer thickness, and preparation protocol. Purified mucin models are therefore most appropriate for mechanistic studies and formulation ranking rather than for direct quantitative prediction of transport through native intestinal mucus.

### 3.2 Native mucus and tissue-based models

Native gastrointestinal mucus provides the highest degree of biochemical fidelity because it contains physiologically assembled mucins together with lipids, ions, proteins, antimicrobial factors, immunoglobulins, cellular material, and microbial products (Lock et al., 2018; Macierzanka et al., 2019). It can be investigated while remaining associated with intestinal tissue or after its collection from the epithelial surface. These two approaches address different experimental questions and should not be considered interchangeable.

Native intestinal tissues can be obtained from laboratory animals, slaughterhouse material, human surgical specimens, or biopsies. Porcine tissues are frequently used because they are readily available and share several anatomical and physiological characteristics with the human gastrointestinal tract. Rodent tissues allow better control over age, diet, disease status, and anatomical site. Nevertheless, mucus thickness, mucin expression, glycosylation, rheology, microbiota, and epithelial permeability differ between animal species and humans (McCoy et al., 2024; Paone and Cani, 2020). Species and anatomical origin must therefore be considered when interpreting translational relevance.

#### 3.2.1 Collection of native mucus

Cell-free native mucus can be collected after gently removing luminal contents and opening the intestinal segment longitudinally. Mucus is generally recovered by scraping, aspiration, or suction from the epithelial surface (Macierzanka et al., 2019). The collected material can then be used in rheological measurements, particle-tracking assays, Transwell inserts, Franz cells, or other diffusion systems.

The collection procedure can substantially influence the properties of the recovered mucus. Extensive washing may remove the loose outer layer, whereas insufficient washing can leave variable quantities of digesta and microbial material. Scraping may disrupt the mucin network, mix mucus originating from different layers, or introduce epithelial cells and tissue fragments. The resulting preparation may retain much of the biochemical composition of native mucus without preserving its original spatial organization (C.-M. Wang et al., 2023).

Fresh mucus should ideally be analyzed immediately. When storage is required, rapid freezing can preserve particle diffusion and microrheological properties more effectively than slow freezing, although freeze–thaw cycles may still alter network connectivity and viscoelasticity (Macierzanka et al., 2019; Witten et al., 2018). Collection method, intestinal region, storage temperature, freezing rate, storage duration, and number of freeze–thaw cycles should consequently be documented.

#### 3.2.2 Chamber-based ex vivo models

Maintaining mucus on the intestinal tissue limits the structural disruption associated with its removal and permits the combined analysis of mucus penetration, epithelial uptake, and transepithelial transport. Ussing chambers are widely used for this purpose (Miyake et al., 2018; Weglinska et al., 2026). An excised intestinal segment is mounted vertically between independent mucosal and serosal compartments. Molecular or particulate transport can be quantified while transepithelial electrical resistance and short-circuit current are measured to monitor barrier integrity and active ion transport.

The independent control of the two compartments enables different apical and basolateral conditions to be established. This is useful for reproducing a poorly oxygenated luminal environment while maintaining tissue oxygenation from the basolateral side. However, tissue viability generally limits experimental duration, and mounting may stretch or damage the epithelium. Washing, dissection, and handling may also remove or redistribute the loosely adherent mucus layer.

#### 3.2.3 Related in situ intestinal models

Ligated intestinal loops and single-pass intestinal perfusion retain the intestinal segment within an anaesthetized animal and are therefore classified as in situ rather than strictly ex vivo models (Xu et al., 2021). They are nevertheless relevant to mucus modelling because they preserve vascular supply and part of the native physiological environment.

In the ligated-loop model, a defined intestinal segment is isolated by ligation and injected with the test formulation. After incubation, the segment can be collected to determine mucus penetration, epithelial uptake, tissue retention, and systemic absorption. The model preserves tissue perfusion but produces a static luminal environment. Ligation may cause local distension and modify fluid balance, mucus secretion, or epithelial physiology.

In single-pass intestinal perfusion, an intestinal segment is cannulated at both ends and perfused at a controlled flow rate (Dahan et al., 2009). Permeability is determined from the disappearance of the compound from the luminal perfusate, its appearance in plasma, or both. This approach provides time-resolved measurements and enables comparisons among intestinal regions. However, luminal disappearance cannot distinguish degradation, mucus retention, epithelial uptake, tissue accumulation, and entry into the circulation. Plasma, tissue, mucus, and histological analyses are therefore required for mechanistic interpretation.

All tissue-based models raise ethical concerns because they depend on animal experimentation or animal-derived materials. They are also affected by interspecies variability and are relatively low-throughput. Slaughterhouse tissues may reduce the number of animals sacrificed specifically for research, but clinical history, feeding status, post-mortem delay, and storage conditions are less controlled. Human specimens provide greater translational relevance but are scarce and commonly obtained from patients with an underlying disease. Tissue-based models are therefore best used for validation after initial screening in more reproducible systems.

### 3.3 Mucus-secreting epithelial cultures

Mucus-secreting cell cultures offer an intermediate level of complexity between cell-free mucus preparations and advanced tissue models. They combine endogenous mucus production with a living epithelial barrier, enabling mucus penetration, epithelial uptake, and transepithelial transport to be studied within the same system. (Lock et al., 2018).

The most established approach is the co-culture of Caco-2 and HT29-MTX cells. Differentiated Caco-2 cells develop an absorptive enterocyte-like phenotype, including apical microvilli and tight junctions between adjacent cells. HT29-MTX cells display a mucus-secreting goblet-like phenotype but generally form a less restrictive epithelial barrier. The relative proportion of both cell types must therefore be optimized to obtain sufficiently homogeneous mucus coverage without excessively reducing epithelial integrity. (Lock et al., 2018).

Mucus production is influenced by cell ratio, passage number, seeding density, culture duration, medium composition, and mechanical stimulation. Submerged, semi-wet, and air–liquid interface cultures can produce different mucus thicknesses and organization. Consequently, the presence of HT29-MTX cells alone does not demonstrate that a physiologically representative mucus barrier has formed. Mucin expression, mucus coverage, thickness, and barrier properties should be characterized experimentally. (Lock et al., 2018).

Epithelial integrity is generally assessed by transepithelial electrical resistance and paracellular permeability markers. Particle mobility within the mucus can be quantified by single- or multiple-particle tracking, which measures individual trajectories, mean-squared displacement, and the proportion of mobile and immobilized particles (Biswas and Levy, 2025). Fluorescence recovery after photobleaching can complement particle tracking for smaller colloids and molecular species. These measurements should be interpreted together with mucus thickness and composition because differences in diffusion may otherwise reflect variation in the model rather than formulation performance.

Cancer-derived Caco-2 and HT29-MTX cells do not reproduce the complete cellular diversity, mucin glycosylation, or disease responsiveness of the human intestine. Moreover, using a Caco-2 monoculture as a mucus-free control introduces differences in cell composition and tight-junction integrity. Removal of mucus using mucolytic agents provides an alternative control but may affect the underlying epithelium. These systems are therefore particularly useful for comparative permeability screening, provided that their cellular and mucus-related limitations are acknowledged.

### 3.4 Organoid-derived and microphysiological models

Intestinal organoids provide a more physiologically representative source of epithelial cells than immortalized cancer-derived lines (López-Jiménez and Mostowy, 2021). They can be generated from adult intestinal stem cells, patient biopsies, embryonic stem cells, or induced pluripotent stem cells and differentiated into enterocytes, goblet cells, Paneth cells, enteroendocrine cells, and other intestinal epithelial populations. They can also retain donor-specific, disease-specific, and gastrointestinal region-specific characteristics.

Three-dimensional organoids reproduce aspects of intestinal epithelial organization but enclose their apical surface within an internal lumen. This limits direct exposure to drugs, nanoparticles, microorganisms, and dietary components. Microinjection (Williamson et al., 2018), fragmentation (Watanabe et al., 2022), or reversal of organoid polarity (Kakni et al., 2022) can provide luminal access, but these approaches are technically demanding and can introduce substantial experimental variability. Dissociating organoids into planar monolayers on permeable supports facilitates independent access to the apical and basolateral compartments while retaining greater cellular diversity than conventional cell lines (Gill et al., 2025).

The integration of organoid-derived cells into gut-on-chip platforms introduces luminal flow, mechanical stimulation, compartmentalization, and controlled oxygen, nutrient, and pH gradients. These factors promote epithelial differentiation and can enhance goblet-cell development and endogenous mucus secretion. Microfluidic devices can additionally include endothelial, immune, neuronal, and stromal cells. Immune components are particularly relevant for studying infection, inflammation, and microbiome-targeted therapeutics because cytokine signalling and immune–microbial interactions regulate both epithelial function and mucus secretion (Özkan et al., 2024; Shin and Kim, 2022).

However, the incorporation of immune cells does not necessarily reproduce mucus as an immunologically active structure. Most current models do not recapitulate the physiological incorporation, spatial distribution, and renewal of secretory immunoglobulins, antimicrobial peptides, complement components, and other immune mediators within the mucus layer. Similarly, interactions among mucin glycans, microbial lectins, immunoglobulins, and epithelial- or immune-derived antimicrobial factors are rarely characterized. Models combining mucus-secreting epithelia with relevant immune cells and microbial communities provide opportunities to investigate cytokine-dependent regulation of mucus production and barrier function. Nevertheless, reconstructing the complete mucus-associated immune environment, including its regional gradients and dynamic response to infection or inflammation, remains an important limitation and an open challenge for the field.

Interestingly, microbiota-containing platforms such as HuMiX physically separate microbial and epithelial compartments while permitting molecular exchange (De Rudder et al., 2026). Other gut-on-chip systems maintain an oxygenated epithelial compartment alongside a microaerophilic or anaerobic luminal environment, enabling co-culture with oxygen-sensitive commensal bacteria (Brasino et al., 2024). These models have demonstrated reciprocal effects between the epithelium and microbiota, including changes in epithelial differentiation, mucus production, microbial composition, and metabolic activity.

The mucus layer is central to these interactions. Mucin glycans, human milk oligosaccharides, and other diet-derived glycans provide selective substrates for microbial metabolism. Microbial degradation of terminal glycans can expose the mucin protein backbone to proteases, while microbial metabolites such as short-chain fatty acids regulate goblet-cell differentiation, immune signalling, and epithelial homeostasis. Mucus should therefore be regarded as an active interface connecting diet, microbial metabolism, immunity, and the response to microbiome-targeted therapeutics.

Most current models nevertheless treat mucus as a single homogeneous layer. Reproduction of the colonic bilayer remains challenging. Simply stacking two mucus formulations may lead to interfacial mixing, sliding, or progressive loss of stratification. A physiologically relevant model should reproduce both a dense, bacteria-restrictive inner layer and a looser, bacteria-permissive outer layer. This requires spatially controlled mucus secretion or deposition, distinct rheological or crosslinking properties, and direct validation of bacterial penetrability in each region. Colon-on-chip systems based on primary or organoid-derived cells currently provide some of the strongest evidence that this organization can emerge through continuous endogenous mucus secretion under flow (Bernheim-Dennery et al., 2026).

Despite their physiological advantages, organoid and microphysiological systems remain costly, technically demanding, and relatively low-throughput. Their performance depends on donor, extracellular matrix, culture medium, device geometry, flow conditions, and experimental duration (De Rudder et al., 2026; Movcana et al., 2026; Porfiryeva et al., 2024; Shah et al., 2016). Model complexity should therefore be determined by the biological question rather than maximized systematically.

## 4 Chemical Engineering of Mucus

The development of experimental models that faithfully reproduce the intestinal mucus barrier has become a central objective in biomaterials science and pharmaceutical research. Early approaches relied heavily on purified mucins extracted from animal tissues, which provided a simplified platform for studying basic properties such as viscosity and diffusion. However, these systems suffer from several limitations, including batch-to-batch variability, partial degradation during extraction, and loss of native structural organization (Howard et al., 2021; Klitgaard et al., 2024). Consequently, their predictive power for in vivo behaviour is often limited.

To address these challenges, researchers have turned to synthetic and semi-synthetic hydrogels designed to mimic the structural and functional characteristics of mucus. Poly (ethylene glycol) (PEG)-based hydrogels have been widely used due to their biocompatibility and tunable properties. By functionalizing PEG chains with mucin-like glycans or charged groups, it is possible to recreate key features such as hydration, viscoelasticity, and electrostatic interactions (Blakeley et al., 2022). Similarly, polysaccharide-based hydrogels, including alginate (Huang et al., 2022), chitosan (Wibel et al., 2021), and hyaluronic acid (Guarise et al., 2023), have been employed to mimic the polyelectrolyte nature of mucus. These materials can be crosslinked through ionic interactions or covalent bonds, allowing precise control over network architecture and mechanical properties.

A particularly promising strategy involves the use of hybrid systems that combine natural mucins with synthetic polymers. These composite materials aim to leverage the biochemical fidelity of mucins while enhancing mechanical stability and reproducibility through synthetic components. For example, a widely explored route involves physical and dynamic crosslinking, where native mucins are combined with boronic-acid–functionalized polymers to form reversible hydrogels under physiological conditions. In these systems, boronic acid moieties interact dynamically with diol groups present on mucin glycans, enabling the formation of adaptable networks whose stiffness and stability can be finely tuned by adjusting the boronic acid content (Nakahata et al., 2022). Importantly, the reversible nature of these interactions allows controlled dissolution of the gel, releasing both mucin and polymer components and thereby providing a means to modulate cell adhesion and biointerfacial properties in a temporally controlled manner. Complementary to this approach, thermoresponsive hybrid systems have been developed by combining mucin with methylcellulose, a polymer known for its temperature-dependent sol–gel transition (Nowald et al., 2016). These formulations remain fluid at room temperature but rapidly form gels at physiological temperature, effectively coupling the selective permeability and biochemical functionality of mucins with the robust and predictable thermal autogelation behaviour of methylcellulose. Such systems are particularly attractive for applications requiring in situ gel formation, including drug delivery and tissue engineering.

In parallel, advances in covalent network formation have enabled the design of more stable and structurally defined mucus mimetics. For instance, silk fibroin–mucin hybrids assembled via tetrazine–norbornene click chemistry provide highly controlled crosslinked architectures, resulting in dual-phase membranes or hydrogels with strong interpolymer adhesion and improved mechanical integrity (Barros et al., 2025). These covalent strategies offer precise control over network topology and durability, which is essential for long-term studies of transport and barrier function. Additionally, efforts to address the inherent mechanical weakness of mucin-based gels have led to the development of reinforced hybrid networks, such as chitosan–mucin hydrogels stabilized with montmorillonite nanoclay and further strengthened through HEMA (2-Hydroxyethyl methacrylate) grafting (Barik et al., 2021). This multi-component approach significantly enhances mechanical strength and resistance to deformation while preserving key mucus-like properties such as hydration and permeability. Collectively, these hybrid material strategies illustrate how combining natural mucins with synthetic or semi-synthetic polymers enables the creation of versatile and tunable platforms that more closely replicate the structural, mechanical, and functional characteristics of native intestinal mucus.

Glycoengineering has emerged as a powerful tool for enhancing the biological relevance of mucus models. Advances in enzymatic glycosylation (Wood et al., 2025) and synthetic glycochemistry (Kohout et al., 2023) have enabled the production of mucin-like glycoproteins with defined glycan structures. This allows researchers to systematically investigate the role of specific glycans in modulating mucus properties and interactions with microbes and drugs. For instance, the incorporation of sialylated glycans has been shown to increase negative charge density and influence nanoparticle diffusion.

## 5 Model selection, characterization, and reporting

Mucus is an important determinant of oral drug absorption and bioavailability (Carlson et al., 2018; Luis and Hansson, 2023a). Its mesh structure produces steric restriction, while electrostatic, hydrogen-bonding, van der Waals, and hydrophobic interactions regulate the retention or diffusion of drugs and particulate formulations. (Carlson et al., 2018) Formulations are consequently designed to display either mucoadhesive or mucus-penetrating properties (Cheng et al., 2021).

On one hand mucoadhesive systems can increase intestinal residence time and create a local drug reservoir, but strong mucus binding may prevent particles from reaching the epithelium and facilitate their elimination during mucus turnover. Mucus-penetrating formulations employ hydrophilic, near-neutral, or zwitterionic surfaces to minimize interactions with mucins and facilitate access to the epithelium (Cheng et al., 2021; Gill et al., 2025). Model selection should therefore reflect whether the intended formulation is designed for local retention, drug release within mucus, or intact carrier transport toward the epithelial surface.

On the other hand multiple-particle tracking (MPT) is one of the most widely used methods for benchmarking the barrier properties of mucus models. By recording the trajectories of fluorescent probe particles, MPT provides information on mean-squared displacement, effective diffusivity, anomalous transport behaviour, and the proportions of mobile and immobilized particles (C.-M. Wang et al., 2023; Wang et al., 2025; D. Wang et al., 2023). However, comparisons between studies require careful standardization because the results depend on particle size, surface chemistry, probe concentration, observation time, tracking algorithm, mucus thickness, temperature, pH, ionic composition, and sample handling. Whenever possible, model validation should include reference particles with well-characterized mucus-penetrating and mucoadhesive behaviours and comparison with freshly collected human mucus or an appropriately justified animal reference. MPT should also be combined with compositional, structural, and rheological characterization, as similar bulk rheological properties do not necessarily imply equivalent microscale transport behaviour.

No single mucus model or method is universally optimal. Purified mucins and synthetic hydrogels are appropriate for initial screening and mechanistic investigations. Biosimilar mucus provides controlled rheological complexity, while mucus-secreting cell cultures permit combined mucus and epithelial permeability measurements. Native tissues are most informative when regional architecture and coupled mucus–epithelium transport are required. Organoid-derived and microphysiological systems are particularly valuable for studying dynamic secretion, disease phenotypes, microbiota, immune components, and patient-specific responses.

A tiered strategy is therefore recommended, beginning with reproducible cell-free models and progressing toward cell-secreted, tissue-associated, or microphysiological systems when increased biological complexity is justified. This approach supports efficient formulation selection while limiting unnecessary animal use (fig. 4).

Beyond biological relevance, the selection of an intestinal mucus model must account for technical complexity, resource requirements, experimental throughput, and validation. Purified mucin dispersions and fully synthetic hydrogels are generally the least resource-intensive approaches (Carlson et al., 2018; Lock et al., 2018). They require standard laboratory equipment, are compatible with relatively rapid preparation and high-throughput screening, and facilitate experimental standardization. However, their biorelevance must be demonstrated by comparison with native mucus in terms of composition, rheology, microstructure, and molecular or particle transport. Native mucus and ex vivo intestinal tissues provide greater compositional and regional relevance but require access to fresh biological material, controlled collection and storage procedures, and, for tissue models, specialized diffusion or perfusion equipment (Özkan et al., 2024). Their throughput is lower, and variability associated with species, anatomical region, health status, and sample handling can increase both experimental complexity and cost.

Mucus-secreting epithelial cultures involve intermediate resource requirements because they require prolonged culture, specialized media, and routine monitoring of epithelial differentiation and barrier integrity. Organoid-derived and microphysiological systems are generally the most resource- and expertise-intensive models because they may require primary-cell expansion, extracellular matrices, microfabricated devices, perfusion equipment, advanced imaging, and trained personnel (Gill et al., 2025). Their increased complexity does not, however, inherently guarantee improved predictive performance. Validation should therefore be adapted to the intended application and include appropriate combinations of mucus thickness and composition, mucin expression, rheological behaviour, multiple-particle tracking, epithelial integrity, permeability to reference compounds, microbial penetrability, and comparison with relevant animal or human data. Because actual monetary costs vary substantially according to local infrastructure, commercial reagents, device configuration, culture duration, and analytical endpoints, the relative resource burden of each model is more informative than a fixed cost estimate.

Finally, the biological and experimental origin of mucus must be reported systematically. At a minimum, studies should specify species, gastrointestinal region, health and feeding status, collection procedure, storage conditions, mucin source, mucus concentration or thickness, pH, ionic composition, and relevant culture or flow conditions. Porcine gastric mucin, bovine submaxillary mucin, rodent intestinal mucus, porcine intestinal mucus, and human cell-secreted mucus should not be treated as interchangeable materials. Standardized reporting and characterization are essential for interpreting differences between studies and improving the predictive value of intestinal mucus models. To provide a practical overview and facilitate fit-for-purpose model selection, the main characteristics, advantages, limitations, and applications of the intestinal mucus models discussed in this section are summarized in Table 1.

**Table 1:** Comparative characteristics and fit-for-purpose applications of experimental intestinal mucus models. The assessments presented are comparative rather than absolute. Model performance depends on mucus origin, gastrointestinal region, preparation and storage conditions, culture protocol, experimental environment, and the intended application.

Model	Hybrid mucin–polymer hydrogels	Gut-on-chip/microphysiological models
Mucus Origine	Purified mucins combined with synthetic or natural polymers	Cell-secreted mucus, often from primary or organoid-derived cells
Reproductibility	Moderate to high	Moderate to low
Glycan Fidelity	Moderate and dependent on mucin source	Moderate to high
Sourcing and Ethics	Partly animal-derived	Depends on cellular source
Scalability	Moderate to high	Low
Physiological relevance	Moderate	High
Principal application	Controlled rheology, diffusion studies and material optimization	Dynamic transport, microbiota, immune interactions and complex barrier studies

## 6 Brief perspective on computational modelling

Computational modelling has emerged as a complementary approach to experimental studies. Molecular dynamics simulations provide insights into the behaviour of mucins at the nanoscale, revealing how glycosylation patterns influence conformation and interactions (Biswas and Levy, 2025; Butenhof and Gerken, 1993). For example, stochastic simulations of mucin-like glycopeptides showed that removal of O-linked carbohydrates decreased both the end-to-end distance and the radius of gyration by approximately one third, supporting the contribution of clustered glycans to the extended conformation of mucins (Butenhof and Gerken, 1993). More recent coarse-grained simulations further indicated that glycan density, glycan charge and interactions with monovalent or divalent cations regulate the expansion and compaction of mucin domains (Biswas and Levy, 2025).

Coarse-grained particle-based models can extend this molecular description to mucus network formation and nanoparticle transport. A dissipative particle dynamics model were developed in which MUC2 and MUC5AC were represented as bead-spring polymers containing hydrophobic protein domains and hydrophilic glycosylated regions (Zhao et al., 2024). The model was used to investigate the effects of mucin composition and concentration on mucus pore size and the diffusion of PEG-coated nanoparticles. Its biorelevance was evaluated by comparison with rheological measurements, experimentally determined pore sizes and single-particle tracking data obtained using mucus secreted by HT29-MTX-E12 cells. Simulated and experimental normalized diffusion coefficients showed similar size-dependent behaviour: particles larger than the average mucus pore size were more likely to become trapped, whereas smaller particles retained greater mobility. However, this model was developed for mucus representative of a human colorectal adenoma environment, and further validation is required before extrapolation to healthy intestinal mucus or other pathological conditions.

At larger scales, continuum models are used to simulate mucus flow and transport phenomena, incorporating parameters such as viscosity, elasticity, and diffusion coefficients. In these approaches, mucus can be treated as a continuous viscous or non-Newtonian medium rather than as an explicit molecular network. For example intestinal mucus was represented as a Herschel-Bulkley fluid and the luminal phase as a Newtonian fluid (Valibeknejad et al., 2023). The continuity and Navier-Stokes equations were coupled with mass-transfer equations to simulate the penetration of Brilliant Blue FCF through a mucus-on-chip configuration. Comparison with experimental observations showed that a diffusion-only model did not adequately reproduce penetration and that convection within the mucus layer had to be included. Sensitivity analysis further demonstrated that both mucus rheology and the diffusion coefficient strongly affected the predicted penetration depth. These models are less computationally demanding than molecular simulations but depend heavily on the accuracy and physiological relevance of their input parameters, boundary conditions and constitutive rheological equations.

Mathematical models can also address biological processes that are particularly difficult to isolate experimentally. Bacterial run-and-tumble motion was combined with antibody diffusion, antibody-bacterium binding and reversible antibody-mucin interactions to examine the ability of IgG and secretory IgA to prevent bacterial penetration through mucus (Xu et al., 2019) et al., 2019). Their model predicted that the accumulation of multiple individually weak IgG-mucin interactions could immobilize antibody-coated bacteria within mucus and that sIgA-mediated agglutination provided only a modest additional contribution under the simulated conditions. This example illustrates how computational modelling can help distinguish competing mechanisms underlying the immunological barrier function of mucus. Nevertheless, such predictions remain sensitive to assumptions regarding mucus thickness, bacterial motility, antigen density, antibody concentration and binding kinetics and therefore require experimental validation.

Artificial intelligence and machine learning are also beginning to play a role in mucus research. These tools can be used to analyse large datasets, identify patterns, and predict system behaviour. Their current contribution is most clearly demonstrated in the analysis of heterogeneous particle trajectories rather than in the direct optimization of mucus hydrogels. The “diffusional fingerprinting" was introduced, in which 17 features describing mean-squared displacement, trajectory geometry, confinement and transitions between diffusional states were extracted from single-particle trajectories (Pinholt et al., 2021). Logistic regression was then used to classify particle populations, while linear discriminant analysis identified the features responsible for their separation. Applied to differently coated nanoparticles moving in mucus, this framework distinguished mucus-inert TPGS-coated particles from unmodified PLGA particles with respective accuracies of approximately 93% and 91%. It also showed that the TPGS coating increased mobility and produced less confined, more Brownian-like transport. A related fingerprinting approach was subsequently applied to simulated nanoparticle trajectories and used multinomial logistic regression to differentiate particles according to size (Zhao et al., 2024). This analysis indicated that larger particles remained trapped for longer periods, while displaying more ballistic behaviour over short observation times.

The resources required for computational approaches depend strongly on model resolution. Molecular and coarse-grained simulations require specialized parameterization and substantial computing time, whereas continuum models are generally less computationally intensive once their geometry, rheological parameters and boundary conditions have been defined. Machine-learning approaches may accelerate the analysis of large trajectory datasets but require sufficiently large, representative and consistently processed training data. In all cases, reduced experimental consumption should not be confused with intrinsic biorelevance. Computational predictions should be validated against independent experimental measurements, including mucus composition and rheology, pore size, multiple-particle tracking, diffusion coefficients, permeability profiles or bacterial penetration.

Looking forward, the integration of experimental and computational approaches will be essential for advancing mucus modelling. Rather than replacing biological models, computational methods can support mechanistic interpretation, sensitivity analysis and experimental design. Their predictive value will depend on transparent reporting of model assumptions and parameters, together with validation using well-characterized intestinal mucus systems.

## 7 Conclusions

Significant progress has been achieved in the development of chemical and biomimetic models of intestinal mucus, reflecting advances in polymer chemistry, glycoscience, and microengineering. The intestinal mucus layer is now recognized as a dynamic and multifunctional hydrogel that plays a central role in host protection, microbial regulation, and molecular transport. Reproducing its structural complexity, particularly the hierarchical organization of mucins and the diversity of glycan patterns, remains a key challenge but also a major driver of innovation in the field. Recent approaches, including synthetic and hybrid hydrogels, have enabled improved control over viscoelasticity, porosity, and charge, enhancing the relevance of in vitro systems for studying drug diffusion and particle transport. In parallel, micro-physiological platforms such as gut-on-chip devices, combined or not with 3D printing strategies, have introduced dynamic and biologically active environments that better recapitulate mucus secretion, host–microbe interactions and drug transport. Computational models further complement these systems by providing multi-scale insights into mucus structure and transport behaviour. Despite these advances, current models still struggle to fully capture the heterogeneity, turnover, and biological responsiveness of native mucus. Future developments will likely focus on integrating chemical precision with biological complexity, including the use of glycoengineered mucins, stimuli-responsive materials, and patient-derived models. The convergence of experimental and computational approaches, supported by emerging data-driven methods, holds strong potential for advancing predictive capabilities. Overall, continued interdisciplinary efforts will be essential to develop next-generation mucus models that bridge the gap between simplified in vitro systems and the in vivo intestinal environment.

## CRediT authorship contribution statement

**Aixa Aguilera-Garrido:** Writing – review & editing, Writing – original draft, Methodology. **Agathe Lemée:** Writing – original draft. **Julien Claudel:** Writing – review & editing, Supervision. **Halima Alem:** Writing – original draft, Visualization, Methodology, Conceptualization.

## Declaration of competing interest

Aixa Aguilera-Garrido, Agathe Lemée, Julien Claudel, and Halima Alem declare that they have no known competing financial interests or personal relationships that could have appeared to influence the work reported in this paper.

## Data Availability

No data was used for the research described in the article.
